# The mGlu_5_ Receptor Protomer-Mediated Dopamine D_2_ Receptor Trans-Inhibition Is Dependent on the Adenosine A_2A_ Receptor Protomer: Implications for Parkinson’s Disease

**DOI:** 10.1007/s12035-022-02946-9

**Published:** 2022-07-12

**Authors:** Wilber Romero-Fernandez, Jaume J. Taura, René A. J. Crans, Marc Lopez-Cano, Ramon Fores-Pons, Manuel Narváez, Jens Carlsson, Francisco Ciruela, Kjell Fuxe, Dasiel O. Borroto-Escuela

**Affiliations:** 1 Department of Cell and Molecular Biology, Science for Life Laboratory, Uppsala Univesity, 75124 Uppsala, Sweden; 2 Pharmacology Unit, Department of Pathology and Experimental Therapeutics, School of Medicine and Health Sciences, Institute of Neurosciences, University of Barcelona, L’Hospitalet de Llobregat, 08907 Barcelona, Spain; 3 Neuropharmacology & Pain Group, Neuroscience Program, Bellvitge Institute for Biomedical Research, IDIBELL, L’Hospitalet de Llobregat, 08907 Barcelona, Spain; 4 Division of Cellular and Molecular Neurochemistry, Department of Neuroscience, Karolinska Institutet, Biomedicum (B0851), Solnavägen 9, 17165 Stockholm, Sweden; 5 Facultad de Medicina, Instituto de Investigación Biomédica de Málaga, Universidad de Málaga, Málaga, Spain; 6 Laboratory of Receptomics and brain disorders, Department of Human Physiology, Faculty of Medicine, University of Malaga, Calle Jiménez Fraud, 10, 29071 Malaga Malaga, Spain

**Keywords:** Adenosine A_2A_ receptor, Dopamine D_2_ receptor, Metabotropic glutamate receptor 5, Heteroreceptor complex, Receptor-receptor interaction, Allosteric modulation

## Abstract

The adenosine A_2A_ receptor (A_2A_R), dopamine D_2_ receptor (D_2_R) and metabotropic glutamate receptor type 5 (mGluR_5_) form A_2A_R-D_2_R-mGluR_5_ heteroreceptor complexes in living cells and in rat striatal neurons. In the current study, we present experimental data supporting the view that the A_2A_R protomer plays a major role in the inhibitory modulation of the density and the allosteric receptor-receptor interaction within the D_2_R-mGluR_5_ heteromeric component of the A_2A_R-D_2_R-mGluR_5_ complex in vitro and in vivo. The A_2A_R and mGluR_5_ protomers interact and modulate D_2_R protomer recognition and signalling upon forming a trimeric complex from these receptors. Expression of A_2A_R in HEK293T cells co-expressing D_2_R and mGluR_5_ resulted in a significant and marked increase in the formation of the D_2_R-mGluR_5_ heteromeric component in both bioluminescence resonance energy transfer and proximity ligation assays. A highly significant increase of the the high-affinity component of D_2_R (D2R_Ki High)_ values was found upon cotreatment with the mGluR_5_ and A_2A_R agonists in the cells expressing A_2A_R, D_2_R and mGluR_5_ with a significant effect observed also with the mGluR_5_ agonist alone compared to cells expressing only D_2_R and mGluR_5_. In cells co-expressing A_2A_R, D_2_R and mGluR_5_, stimulation of the cells with an mGluR_5_ agonist like or D_2_R antagonist fully counteracted the D_2_R agonist-induced inhibition of the cAMP levels which was not true in cells only expressing mGluR_5_ and D_2_R. In agreement, the mGluR_5_-negative allosteric modulator raseglurant significantly reduced the haloperidol-induced catalepsy in mice, and in A_2A_R knockout mice, the haloperidol action had almost disappeared, supporting a functional role for mGluR_5_ and A_2A_R in enhancing D_2_R blockade resulting in catalepsy. The results represent a relevant example of integrative activity within higher-order heteroreceptor complexes.

**Supplementary Information:**

The online version contains supplementary material available at 10.1007/s12035-022-02946-9.

## Introduction

The first pieces of evidence for antagonistic glutamate receptor with dopamine D_2_ receptor (D_2_R) interactions were found in 1983–1984 through the ability of glutamate to reduce the affinity of the high-affinity D_2_R agonist binding sites in striatal membrane preparations. Subsequently, it was observed that mGluR_5_ agonists alone or combined with an A_2A_R agonist (CGS-21680) can reduce the affinity of the high-affinity state of D_2_R for agonist binding sites in the rat striatum [1]. Co-immunoprecipitation experiments also indicated the existence of A_2A_R-mGluR_5_ heteroreceptor complexes in HEK293 cells and rat striatal membrane preparations [2]. The colocation of the receptors in striatal neurons was demonstrated [3, 4] as well as their synergistic interactions as studied with in vivo microdialysis and intracellular signalling in striatal preparations [2, 5, 6].

In 1974, the discovery that the methylxanthines caffeine and theophylline could enhance the contralateral turning behaviour induced by levodopa and dopamine receptor agonists in the hemi-Parkinsonian rat model was one early finding leading to the hypothesis that antagonistic adenosine-dopamine interactions existed [7, 8]. Today, a considerable amount of molecular and functional experimental data supports the view that A_2A_R and D_2_R form heteroreceptor complexes with antagonistic receptor-receptor interactions on the plasma membrane [9–16].

The existence of A_2A_R-D_2_R-mGluR_5_ higher-order oligomers was postulated, and it was proposed that the receptor-receptor interactions within this high-order complex are important to modulate the dorsal and ventral striatal-pallidal GABA neurons [2, 3, 8]. Years later, it was proposed that combined treatment with A_2A_R and mGluR_5_ agonists targeting A_2A_R-D_2_R-mGluR_5_ heteroreceptor complexes in the ventral striatal-pallidal GABA pathway can represent a new strategy for the treatment of schizophrenia [17]. Also, the combine treatment with selective A_2A_ and mGluR_5_ receptor antagonists represents an alternative therapeutic approach to Parkinson’s disease [18–20].

A combination of bimolecular fluorescence complementation assays and bioluminescence resonance energy transfer assays as well as the sequential resonance energy transfer technique was used to show that A_2A_R-D_2_R-mGluR_5_ heteroreceptor complexes exist in living cells [21]. In addition, high-resolution immunoelectron microscopy was also used to further demonstrate their existence in striatal glutamate synapses [21]. An integrative role of these receptor complexes in adenosine, dopamine and glutamate transmission was also proposed [8, 22, 23]. Recently, A_2A_R, D_2_R and mGluR_5_ receptor-receptor interactions were also found to modulate the activity of the striatal-pallidal GABA neurons based on in vivo dual-probe microdialysis [24].

Herein, new findings that further expand the understanding of A_2A_R-D_2_R-mGluR_5_ heteroreceptor complexes are presented. Results in cellular models first demonstrated that A_2A_R promotes the D_2_R and mGluR_5_ receptor-receptor interactions, and its participation increases the density of the D_2_R-mGluR_5_ heterocomplexes. Binding and functional experiments indicated that A_2A_R and mGluR_5_ upon agonist activation play a significant role in modulating the composition, density and signalling of A_2A_R-D_2_R-mGluR_5_ heteroreceptor complexes. This was also observed in A_2A_R or D_2_R knockout mice when studying the effects of the mGluR_5_ negative allosteric modulator raseglurant on locomotor activity.

## Methods

### Plasmid Constructs

The cDNA encoding the rat mGluR_5_ was cloned (without stop codon) in pGFP^2^-N1 vector (PerkinElmer, Waltham, MA, USA) using standard molecular biology techniques. The D_2_R^*Rluc*^ construct used has been described previously in Borroto-Escuela et al. 2010 [25].

### Drugs and Chemicals

The A_2A_R agonist 4-[2-[[6-Amino-9-(*N*-ethyl-β-D-ribofuranuronamidosyl)-9*H*-purin-2-yl]amino]ethyl]benzenepropanoic acid hydrochloride (CGS-21680), the selective A_2A_R antagonist 4-(2-[7-Amino-2-(2-furyl)[1,2,4]triazolo[2,3-*a*][1,3,5]triazin-5-ylamino]ethyl)phenol (ZM-241385), the mGluR_5_ agonist (*RS*)-2-Chloro-5-hydroxyphenylglycine sodium salt (CHPG), the mGluR_5_ antagonist 2-Methyl-6-(phenylethynyl)pyridine hydrochloride (MPEP) and the D_2_R antagonist 4-[4-(4-Chlorophenyl)-4-hydroxy-1-piperidinyl]-1-(4-fluorophenyl)-1-butanone hydrochloride (haloperidol) were purchased from Tocris Bioscience (UK), and the mGluR_5_ negative allosteric modulator 2-[(3-Fluorophenyl)ethynyl]-4,6-dimethyl-3-pyridinamine hydrochloride (raseglurant) was purchased from Hello Bio (Republic of Ireland). The concentrations of CGS-21680 (100 nM) and ZM-241385 (1 μM) were chosen in agreement with our previous studies [26, 27]. The concentrations of CHPG (500 nM) and MPEP (300 nM) have been selected on the basis of previous studies suggesting that, in this concentration range, the compounds selectively act as agonist or antagonist of mGluR_5_, respectively [18, 24, 28, 29]. Finally, the dose of haloperidol (1 mg/kg) and raseglurant (1 mg/kg) used in mouse behavioural experiments was previously described [30, 31]. Also, isobutyl-1-methylxanthine (IBMX) and 4-(3-butoxy-4-methoxybenzyl) imidazolidone (Ro 20-1724) were purchased from Tocris Bioscience (Bristol, UK).

### Cell Culture and Transfection

Human embryonic kidney 293T (HEK293T cells (American Type Culture Collection, Manassas, VA, USA) cells were grown in Dulbecco’s Modified Eagle’s Medium supplemented with 2 mM L-glutamine, 100 units/ml penicillin/streptomycin and 10% (v/v) foetal bovine serum at 37 °C in an atmosphere of 5% CO_2_. Cells were plated in 6-well plates (1 × 10^6^cells/well), 96-well plates (1 × 10^4^cells/well) or in 75 cm^2^ flasks and cultured overnight prior to transfection or experimental procedures. Cells were transiently transfected using linear polyethyleneimines (Polysciences Inc., Warrington, PA, USA) according to the manufacturer’s instructions.

### Animals

A_2A_R^−/−^ and D_2_R^−/−^ mice generated on a CD-1 genetic background [30, 32] and the corresponding wild-type littermates weighing 20–25 g were used. The animal protocol (no. 7085) was approved by the University of Barcelona Committee on Animal Use and Care. Animals were housed and tested in compliance with the guidelines provided by the Guide for the Care and Use of Laboratory Animals [33] and following the European Union directives (2010/63/EU), the ARRIVE guidelines [34]. Mice were housed in groups of five in standard cages with access to food and water ad libitum while maintained under a 12-h dark/light cycle (starting at 7:30 AM), 22 °C temperature and 66% humidity (standard conditions). All animal experimentation was carried out in a period comprehended between 9:00 AM and 6:00 PM by a researcher blind to drug treatments.

### Locomotor Activity Tests

Mice spontaneous or drug-induced locomotor activity was assessed by the open field test. In brief, animals were administered intraperitoneal (i.p.) with raseglurant (1 mg/kg) or vehicle-saline with 5% DMSO and 5% Tween 20 30 min before the testing session. Non-habituated mice were placed in the centre of an activity field arena (30 × 30 cm, surrounded by four 50-cm-high black-painted walls) equipped with a camera above to record activity and connected to the light source. The total distance travelled was analysed using SPOT tracker function from ImageJ (NIH, Bethesda, MD, USA), as previously described [30].

### Catalepsy Test

Mouse catalepsy was induced by the administration (i.p.) of haloperidol (1 mg/kg) [30]. After 1 h, haloperidol-induced catalepsy was measured as the duration in seconds of an abnormal upright posture in which the forepaws of the mouse were placed on a horizontal wooden bar (0.6 cm of diameter) that was located 4.5 cm above the floor. Subsequently, mice were administered (i.p.) with either vehicle (i.e. saline with 5% DMSO and 5% Tween) or raseglurant (1 mg/kg). After 20 min, a second haloperidol-induced catalepsy measurement was performed.

The rationale for the use of raseglurant (a mGluR5-negative allosteric modulator) instead of a full antagonist was based on the theoretical advantages that allosteric modulators offer compared with their competitive counterparts. mGluR5 allosteric modulators (negative allosteric modulators (NAM) and positive allosteric modulators (PAM)) have the potential for greater subtype selectivity when compared to orthosteric ligands. Also, mGluR5 NAM and PAM do not possess intrinsic activity and are assumed to be quiescent in the absence of an endogenous agonist and only modulate receptor function when the endogenous agonist is present. In this manner, NAM and PAM have the potential to retain spatial and temporal aspects of endogenous receptor signalling. This is of particular interest for CNS targets where optimal neurotransmission is likely to have an improved therapeutic outcome as opposed to sustained receptor blockade or activation.

### Haloperidol-Induced Catalepsy

Mice (*n* = 10) were randomly assigned to treatment groups, and behavioural testing was performed blind to treatment. The dopamine D_2_ receptor (D_2_R) antagonist, haloperidol (1 mg/kg, s.c.), was administered to induce catalepsy. Thirty minutes after the haloperidol administration, mice experienced a full cataleptic response. At this time point, for each mouse, the state of catalepsy was tested by gently placing their front limbs over an 8-cm-high horizontal bar. The intensity of catalepsy was assessed by measuring the time the mice remain in this position being completely immobile for a maximum of 120 s. Only mice that remained cataleptic for the entire 120 s were used for subsequent drug testing. After 30 min of the baseline measurement vehicle (0.5% methylcellulose and 2% DMSO), PBF509 was administered orally via gavage (3, 10 or 30 mg/kg, p.o.), and the catalepsy was then determined at 15, 30 and 60 min PBF509 administration. For each time point, the number of responding mice and the total cataleptic time for each animal were determined.

### Membrane Preparation

HEK293T cells or mouse striata were homogenized in ice-cold 10 mM Tris HCl, pH 7.4, 1 mM EDTA and 300 mM KCl buffer containing a protease inhibitor cocktail (Roche, Penzberg, Germany) using a Polytron for three periods of 10 s each. The homogenate was centrifuged for 10 min at 1000 × *g*. The resulting supernatant was centrifuged for 30 min at 12,000 × *g*. The membranes were dispersed in 50 mM Tris HCl (pH 7.4) and 10 mM MgCl_2_, washed and resuspended in the same medium. Protein concentration was determined using the BCA protein assay kit (Thermo Fisher Scientific, Inc., Rockford, IL, USA).

### Bioluminescence Resonance Energy Transfer Saturation Assay

BRET^2^ saturation curves have been particularly used with the aim to establish the oligomeric order of receptor complexes, as well as the proportion of receptors engaged in dimers or oligomers (BRETmax). In the current work, bioluminescence resonance energy transfer (BRET^2^) saturation assays were carried out using plasmids encoding for D_2_R^*Rlu*c^ and mGluR_5_^*GFP2*^ according to previously published methods [9, 26, 35, 36]. The netBRET^2^ ratio was defined as the BRET ratio for co-expressed Rluc and GFP^2^ constructs normalized against the BRET ratio for the Rluc expression construct alone: netBRET^2^ ratio = [(GFP^2^ emission at 515 ± 30 nm)/(Rluc emission 410 ± 80 nm)]-cf. The correction factor, cf, corresponds to (emission at 515 ± 30 nm)/(emission at 410 ± 80 nm) found with the receptor-Rluc construct expressed alone in the same experiment. The maximal value of BRET (netBRET^2^max) corresponds to the situation when all available donor molecules are paired up with acceptor molecules [8]. Also, saturation assay was used to compare the relative affinity of receptors for each other and their probability to form a complex, the so-called BRET50, which represents the acceptor/donor ratio giving 50% of the maximal signal. The ratio is calculated from fluorescence and bioluminescence values expressed as arbitrary units. BRET50 values should not be regarded as a common or classical value to expressed affinities as Molar units. Pairs with low BRET_50_ value thought to form oligomers or an increased tendency to dimerize, while high BRET_50_ values indicate weak interaction or the absence of interaction between the investigated receptors. The specificity of D_2_R^*Rluc*^-mGluR_5_^*GFP2*^ interactions was assessed by comparison with co-expression of A_1_R^*GFP2*^ and D_2_R^*Rluc*^.

### In Situ PLA in Cultured Cells

In situ proximity ligation assay (PLA) in cultured cells was performed using the Duolink in situ PLA detection kit (Sigma-Aldrich, St. Louis, MO, USA), following the protocol described previously [11, 37, 38] using mouse monoclonal anti-D_2_R (2 μg/ml, MABN53; Millipore, Billerica, MA, USA) and rabbit polyclonal anti-mGluR_5_ (2 μg/ml, AB5675; Millipore) primary antibodies. PLA control experiments employed only one primary antibody. The PLA signal was visualized and quantified by using a TCS-SL confocal microscope (Leica Lasertechnik GmbH, Heidelberg, Germany) and the Duolink ImageTool software. High magnifications of the microphotograph were taken and visualized using multiple z-scan projections.

The background signal was estimated from both PLA control experiments and from PLA experiments performed on non-transfected HEK293T cells (HEK293T cell line expresses endogenously small amount of D_2_R, A_2A_R and mGluR_5_). In general, the positive PLA values obtained in these experiments were residuals. The assay cut-off value was set to two standard deviations over the background signal. Therefore, samples with values below this cut-off were negative for the interaction of interest, while samples with values higher than the threshold were positive.

### Immunohistofluorescence and In Situ PLA in Mouse Brain

Mice were anaesthetized and intracardially perfused with 50–200 ml of ice-cold 4% formaldehyde solution (Sigma-Aldrich, St. Louis, MO, USA) in phosphate-buffered saline (PBS; 1.47 mM KH_2_PO_4_, 8.07 mM Na_2_HPO_4_, 137 mM NaCl, 0.27 mM KCl with pH 7.2). The brains were post-fixed overnight in the same 4% formaldehyde solution at 4 °C. The vibratome (Leica Lasertechnik GmbH, Heidelberg, Germany) was used to make coronal section (50 μm). Slices were collected and kept in Walter’s antifreezing solution (30% glycerol, 30% ethylene glycol in PBS with pH 7.2) at −20 °C until further processing [39].

For immunohistofluorescence (IHF), experiments coronal brain slices were washed three times with PBS for 10 min at 22 C, then permeabilized with 0.3% Triton X-100 in PBS (2 h at 22 °C) and rinsed (3×) with washing solution (PBS containing 0.05% Triton X-100, 10 min, at 22 °C). Blocking of the slices was performed with washing solution containing 10% normal donkey serum (NDS; Jackson ImmunoResearch Laboratories, Inc., West Grove, PA, USA) for 2 h at 22 °C. To avoid unspecific binding, the slices were incubated with secondary anti-mouse IgG (no. 715-005-150; Jackson ImmunoResearch Laboratories, Inc., West Grove, PA, USA) in washing solution (2 h at 22 °C). Then, the slices were incubated with mouse anti-mGluR_5_ monoclonal (20 μg/ml, MABN540; Millipore) and rabbit anti-D_2_R polyclonal (1 μg/ml, D_2_R-Rb-Af960; Frontier Institute Co. Ltd, Shinko-nishi, Ishikari, Hokkaido, Japan) in washing solution with 5% NDS overnight at 4 °C. Subsequently, the slices were washed twice with a washing solution containing 1% NDS (10 min at 22 °C). Next, the slices were incubated with anti-Cy2 donkey anti-rabbit (1:200; Jackson ImmunoResearch Laboratories, West Grove, PA, USA) and anti-Cy3 donkey anti-mouse (1:200; Jackson ImmunoResearch Laboratories, West Grove, PA, USA) in washing solution with 1% NDS for 2 h at 22 °C. Finally, slices were washed two times with washing solution containing 1% NDS (10 min at 22 °C), two times with PBS (10 min at 22 °C) and then mounted with Duolink^®^ in situ mounting medium with DAPI (Sigma-Aldrich). The Leica TCS 4D confocal scanning laser microscope (Leica Lasertechnik GmbH, Heidelberg, Germany) was used to capture the fluorescence striatal images.

For in situ PLA in mouse brain, the Duolink in situ PLA detection kit (Sigma-Aldrich) was used as previously described [37, 39, 40]. Thus, the experimental procedure until the secondary antibody incubation step was the same as the IHF (see above). Subsequently, the following steps were performed according to the manufacturer’s protocol. Images were acquired and analysed as previously described [39]. The background signal was estimated from PLA control experiments, and the assay cut-off value was performed as described above.

### Radioligand Competition Binding Experiments

For the binding experiments, membrane preparations (60 μg protein/ml) were obtained from HEK293T cells expressing either D_2_R and mGluR_5_ or A_2A_R, D_2_R and mGluR_5_, and [^3^H]-raclopride (Novandi Chemistry AB, Södertälje, Sweden) competition assays with minor modifications were performed according to previously published methods [26, 27, 41]. [^3^H]-raclopride (75 Ci/mmol), a D_2_-like receptor antagonist competing [42] with quinpirole for binding to D_2_-like receptors in HEK293T membrane preparations, was used to determine the D_2_R high-affinity (*K*_i, *High*_) and D_2_R low-affinity (*K*_i, *Low*_) values. (+)-Butaclamol, a selective D2R antagonist (100 μM, Sigma-Aldrich), was used to determine the non-specific binding. The amount of bound [^3^H]-raclopride was determined by liquid scintillation spectrometry.

### cAMP Functional Assay

Intracellular cAMP levels were determined using a cAMP-Glo™ assay detection kit (Promega, Madison, WI, USA). HEK293T cells expressing either D_2_R and mGluR_5_ or A_2A_R, D_2_R and mGluR_5_ were plated at a density of 10,000 cells/well in 96-well microtiter plates coated with poly-L-lysine (Sigma-Aldrich) and incubated overnight. Culture medium was then removed; cells were washed with 1 × PBS before the induction buffer (red phenol/serum-free DMEM containing 500 μM IBMX and 100 μM Ro 20-1724) was added. The cells were incubated for 1 h prior to drug incubation. To examine the *G*_i_ protein-mediated inhibition of adenylyl cyclase, the levels of cAMP were first raised with 5 µM forskolin for 10 min. Drug dilutions were prepared in the induction buffer, and the temperature- and carbon dioxide-equilibrated drug dilutions (37 °C cell culture incubator for 30 min) were added as indicated, and cells were then incubated at 37 °C for 30 min. The assay was performed accordingly to the manufacturer’s specifications (Promega, Sweden). Readings of luminescence intensity were performed using the POLARstar Optima plate reader (BMG Lab Technologies, Offenburg, Germany). cAMP levels in non-transfected, non-treated cells and non-transfected cells treated only with forskolin were defined as basal and control, respectively.

### Gel Electrophoresis and Immunoblotting

Sodium dodecyl sulphate polyacrylamide gel electrophoresis (SDS/PAGE) was performed using 7% polyacrylamide gels. Proteins were transferred to Hybond-LFP polyvinylidene difluoride (PVDF) membranes (GE Healthcare, Chicago, IL, USA) using the Trans-Blot Turbo™ transfer system (Bio-Rad, Hercules, CA, USA) at 200 mA/membrane for 30 min. PVDF membranes were blocked with 5% (wt/vol) dry non-fat milk in phosphate-buffered saline (PBS; 8.07 mM Na_2_HPO_4_, 1.47 mM KH_2_PO_4_, 137 mM NaCl, 0.27 mM KCl, pH 7.2) containing 0.05% Tween-20 (PBS-T) during 1 h at 20 °C before being immunoblotted with the indicated antibody in blocking solution overnight at 4 °C. PVDF membranes were washed with PBS-T three times (5 min each) before incubation with either a HRP-conjugated rabbit anti-mouse IgG (1/10,000) or HRP-conjugated goat anti-rabbit IgG (1/30,000) in blocking solution at 20 °C during 2 h. After washing the PVDF membranes with PBS-T three times (5 min each), the immunoreactive bands were developed using a chemiluminescent detection kit (Thermo Fisher Scientific) and detected with an Amersham Imager 600 (GE Healthcare Europe, Barcelona, Spain).

### Statistical Analysis

The number of independent experiments (*n*) in each group is indicated in figure legends. Data are represented as mean ± standard error of mean (SEM). Outliers were assessed by the ROUT method [43]; thus, subjects were excluded assuming a *Q*-value of 1% in GraphPad Prism 9 (San Diego, CA, USA). Data normality was assessed by the Shapiro-Wilk normality test (*p* < 0.05). When two groups were evaluated, unpaired Student’s *t*-test or Mann-Whitney *U*-test was used. Comparisons among more than two experimental groups were performed by one-, two- or three-way factor analysis of variance (ANOVA) followed by either Dunnett’s, Šídák’s or Tukey post hoc test using GraphPad Prism 9, as indicated in the figure legends. A *p*-value ≤ 0.05 was considered significant.

#### Results

### *BRET*.^*2*^* Experiments: Transient Co-expression of A*_*2A*_*R with D*_*2*_*R and mGluR*_*5*_* Had a Significant Impact on D*_*2*_*R-mGluR*_*5*_* Heteroreceptor Complex Formation*

**Fig. 1:**
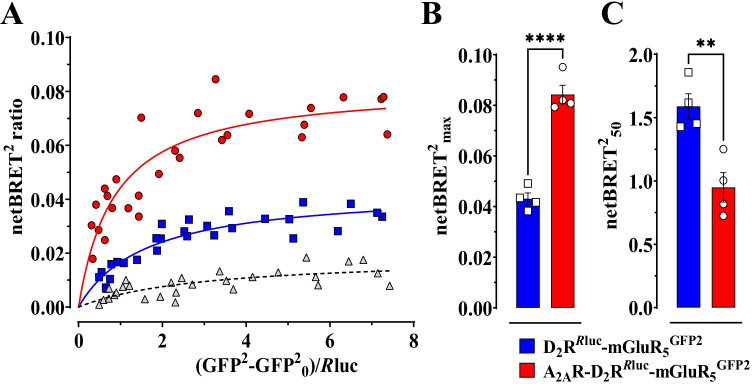
Effect of A_2A_R expression in D_2_R-mGluR_5_ heteromer formation assessed by BRET^2^ assay in HEK293T cells. Cells were transiently transfected with plasmids encoding the D_2_R tagged with *R*luc (i.e. D_2_R^*R*luc^) and mGluR_5_ with GFP2 (i.e. mGluR_5_^GFP2^) in the absence (blue squares) or presence (red circles) of A_2A_R expression. The A_1_R^GFP2^–D_2_R^*R*luc^ pair was used as a control (grey triangles). BRET^2^ saturation curves (**A**) were constructed by co-transfecting a constant amount of the plasmid for D_2_R^*R*luc^ and increasing amounts of the mGluR_5_^GFP2^ plasmid or A_1_R^GFP2^ plasmid. Curves are based on mean values of four independent experiments performed in quadruplicates. The netBRET^2^_max_ (**B**) and netBRET^2^_50_ (**C**) values from the BRET.^2^ saturation curves shown in **A** are represented. BRET ratio is calculated from fluorescence and bioluminescence values expressed as arbitrary units. Results are expressed as mean ± SEM (*n* = 4, each determination performed in quadruplicates). ****p < 0.0001 and **p < 0.01, Student’s *t*-test

HEK293T cells were transiently transfected with constant amounts of D_2_R^*Rluc*^ and increasing amounts of plasmids encoding for mGluR_5_^*GFP2*^ with/without transient co-expression of A_2A_R. The transient co-expression of A_2A_R with D_2_R^*Rluc*^ and mGluR_5_^*GFP2*^ had a significant impact on D_2_R^*Rluc*^-mGluR_5_^*GFP2*^ heteroreceptor complex formation (Fig. 1A). Transient co-expression of A_2A_R promoted a significant increase of netBRET^2^max ratio value (0.084 ± 0.003 AU) compared to that found in cells without transient co-expression of A_2A_R (0.043 ± 0.002 AU) (Fig. 1B). When the A_2A_R was coexpressed with D_2_R^*Rluc*^ and mGluR_5_^*GFP2*^, these receptors hence showed an increased ability to heteromerize.

Also, saturation assay was used to compare the relative affinity of receptors for each other and their probability to form a complex, the so-called BRET50, which represents the acceptor/donor ratio giving 50% of the maximal signal. The netBRET^2^50 ratio value for D_2_R^*Rluc*^-mGluR_5_^*GFP2*^ heteromerization was significantly reduced by transient co-expression of A_2A_R from (1.58 ± 0.09 AU) to (0.94 ± 0.11 AU) (Fig. 1C) indicating increased affinity of the two receptor protomers for each other. Pairs with low BRET_50_ value thought to form oligomers or an increased tendency to dimerize, while high BRET_50_ values indicate weak interaction or the absence of interaction between the investigated receptors.

### *Proximity Ligation Assay Experiments: Transient Co-expression of A*_*2A*_*R Promoted the Formation D*_*2*_*R-mGluR*_*5*_* Heteroreceptor Complexes in HEK Cells*

**Fig. 2:**
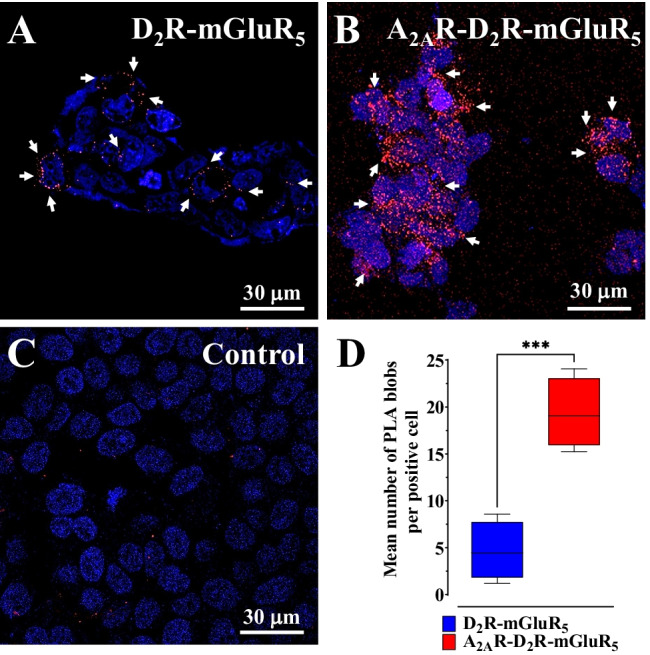
In situ PLA assessment of D_2_R-mGluR_5_ heteromer formation in the absence (**A**) or presence (**B**) of A_2A_R (see “Methods”). The in situ PLA-positive D_2_R-mGluR_5_ heteroreceptor complexes were shown as red blobs (arrows) and nuclei in blue (DAPI staining). A negative in situ PLA control (**C**) was included by incubating the cells in the absence of the primary anti-D_2_R antibody. **D** Quantification of D_2_R-mGluR_5_ complexes. The number of PLA blobs (red clusters) per positive cell (*n* = 4 × 50 cells) was assessed as described in Methods. Results were expressed as mean ± SEM (*n* = 4 independent experiments). ****p < 0.0001 and **p < 0.01, Student’s *t*-test

The role of A_2A_R in the dynamics of the D_2_R-mGluR_5_ heteromers was also evaluated by in situ proximity ligation assays (PLA) in transiently co-transfected HEK293T cells. The PLA results were in line with the results from the BRET^2^ assays. The in situ PLA demonstrated the existence of D_2_R-mGluR_5_ heteroreceptor complexes in cells to a low degree without transient co-expression of A_2A_R (Fig. 2A). Furthermore, the transient co-expression of A_2A_R highly significantly promoted the formation D_2_R-mGluR_5_ heteroreceptor complexes as shown by the marked increase in the number of PLA-positive D_2_R-mGluR_5_ complexes, while this was significantly reduced in HEK293T cells without co-expressing A_2A_R (Fig. 2 B and D). Few and weak PLA clusters were detected in the PLA-negative controls (lack of D_2_R antibodies) representing background labelling (Fig. 2C).

**Fig. 3:**
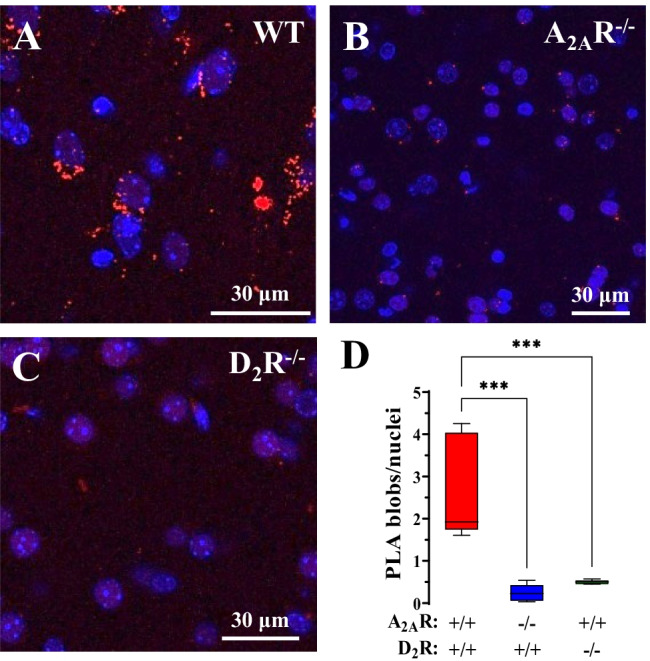
Assessment of D_2_R-mGluR_5_ heteromer formation in mouse dorsal striatum by in situ PLA. Photomicrographs showing PLA recognition of D_2_R-mGluR_5_ heteromers in the dorsal striatum of wild type (**A**), A_2A_R^–/–^ (**B**) and D_2_R^–/–^ (**C**) mice. The in situ PLA-positive D_2_R-mGluR_5_ heteroreceptor complexes are shown as red blobs (arrows) and nuclei in blue (DAPI staining). **D** Quantification of D_2_R-mGluR_5_ complexes showing a highly significant reduction of D_2_R-mGluR_5_-positive red blobs in the absence of A_2A_R^–/–^ or D_2_R^–/^.^–^. The number of PLA blobs (red clusters) per nucleus was assessed as described in Methods. Results were expressed as mean ± SEM (*n* = 5 animals). ***p < 0.001, one-way ANOVA followed by Dunnett’s post hoc test when compared with wild-type animals

The specificity of the PLA-positive D_2_R-mGluR_5_ complexes, shown as red blobs in the mouse dorsal striatum (Fig. 3A), was demonstrated using D_2_R^−/−^ mice (Fig. 3C). In the sections from the mouse striatum, the appearance of the red PLA-positive D_2_R-GluR_5_ complexes, shown as mean number of red blobs/Nucleus, was markedly and highly significantly reduced (Fig. 3D). Furthermore, the loss of the red D_2_R-mGluR_5_ blobs to the same high degree in the A_2A_R^−/−^ mice (Fig. 3 B, D) likely reflects the requirement of D_2_R-mGluR_5_ heterocomplexes to be part of an A_2A_R-D_2_R-mGluR_5_ to be expressed in the mouse striatum, probably by dorsal striatal-pallidal GABAergic neurons. In this way, it forms D_2_R-mGluR_5_ complexes that are close enough to be visualized by PLA.

### *[*.^*3*^*H]-Raclopride/Quinpirole Competition Experiments: the A*_*2A*_*R and mGluR*_*5*_* Protomers Interact and Modulate D*_*2*_*R Protomer Recognition*

**Fig. 4:**
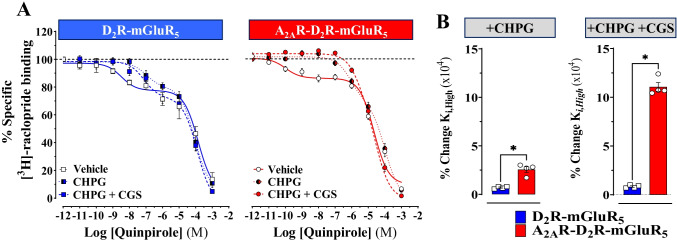
Assessing A_2A_R-dependent allosteric modulation of D_2_R-GluR_5_ heteromer by [^3^H]-raclopride/quinpirole competition binding experiments. **A** Competition assays were performed in HEK293T cells transiently expressing D_2_R and mGluR_5_ (blue squares) or A_2A_R, D_2_R and mGluR_5_ (red circles) with/without agonist(s)/antagonist(s) for adenosine A_2A_R or mGluR_5_ either alone or in combination as indicated. ( +)-Butaclamol (100 μM) was used to determine the non-specific binding, and the specific binding at the lowest concentration of the [^3^H]-raclopride employed was defined as 100%. Results are expressed as percentage of specific binding (mean ± SEM; *n* = 4 independent experiments performed in triplicate). **B** Percentage of change comparing CHPG alone or CHPG plus CGS-21680-induced changes in the D_2_R high affinity values ((*K*_i, High_ (nM)) with/without transient co-expression of A_2A_R. Results are expressed as means ± S.E.M.; *n* = 4, each determination performed in triplicate. *p < 0.05, Mann–Whitney *U*-test

In HEK293T cells expressing D_2_R and mGluR_5_, the mGluR_5_ agonist CHPG (500 nM) reduced the affinity of the high-affinity state (*K*_i, *High*_) of the D_2_R for the agonist quinpirole with no effects on its low-affinity state (*K*_i_, _*Low*_). Co-treatment with A_2A_R agonist CGS-21680 (100 nM) did not significantly alter the D_2_R *K*_i, *High*_ and *K*_i, *Low*_ values obtained when the cells were treated only with CHPG (500 nM) (Fig. 4A and Table 1). In HEK293T cells expressing A_2A_R, D_2_R and mGluR_5_, mGluR_5_ agonist stimulation also reduced the affinity of the high-affinity state (*K*_i, *High*_) of the D_2_R for the agonist quinpirole with no statistically significant effects on its low-affinity state (*K*i, _*Low*_) (Fig. 4A and Table 2). However, the transient co-expression of A_2A_R by itself (without agonist stimulation) potentiates mGluR_5_ agonist effects on the high-affinity D_2_R agonist binding sites (Fig. 4B, Tables 1 and 2). Finally, the co-stimulation of A_2A_R and mGluR_5_ synergistically increased in the *K*_i, *High*_ values of the D_2_R protomer upon co-expression of the A_2A_R (Table 2). Nevertheless, in cells expressing A_2A_R, D_2_R and mGluR_5_, further analysis should be performed to test the effect of combine treatment of A_2A_R (ZM-241385) and mGluR_5_ (CHPG) to figure out if the expression of A_2A_R, without agonist stimulation and its corresponding constitutive activity, is responsible for increased in the *K*_i, *High*_ values of the D_2_R protomer upon co-expression of the A_2A_R.

**Table 1:** Values for quinpirole binding site affinities to the D_2_-likeR by [.^3^H]-raclopride/quinpirole competition assays in HEK293T cells transiently expressing D_2_R and mGluR_5_ incubated with agonist(s) or/and antagonist(s) as indicated

	***K***_**i, *High***_*** (nM)***	***K***_**i, *Low***_*** (nM)***
+ Vehicle	1.1 ± 0.4	155 ± 73
+ CHPG	83 ± 7***	166 ± 63
+ CHPG + CGS-21680	99 ± 9***	283 ± 179
+ CHPG + MPEP	5.6 ± 2^§§§^	40 ± 18
+ CHPG + MPEP + CGS-21680 + ZM-241385	16 ± 5^†††^	63 ± 19
+ CGS-21680	4.3 ± 2.2	127 ± 62
+ CGS-21680 + ZM-241385	5.7 ± 0.9	21 ± 62

*K*_i, *High*_, D_2_R high-affinity value and *K*_i, *Low*_, D_2_R low-affinity value. Data are means ± SEM; *n* = 4, each determination performed at least in triplicate. Statistical analysis was performed by one-way ANOVA followed by the Tukey post hoc test. ***(*p* < 0.001); *significant increased* compared to vehicle. §§§(*p* < 0.001); *significant reduced* compared to cells incubated with CHPG. †††(*p* < 0.001); *significant reduced* compared to cells incubated with CHPG and CGS-21680

**Table 2:** Values for quinpirole binding site affinities to the D_2_-likeR by [.^3^H]-raclopride/quinpirole competition assays in HEK293T cells transiently expressing A_2A_R, D_2_R and mGluR_5_ incubated with agonist(s) or/and antagonist(s) as indicated

	***K***_**i, *High***_*** (nM)***	***K***_**i, *Low***_*** (nM)***
+ Vehicle	1.6 ± 1	45 ± 18
+ CHPG	411 ± 46***	106 ± 41
+ CHPG + CGS-21680	1781 ± 72***, §§§	49 ± 14
+ CHPG + MPEP	3.4 ± 1.7§§§	19 ± 6
+ CHPG + MPEP + CGS-21680 + ZM-241385	43 ± 10†††	158 ± 65
+ CGS-21680	743 ± 53***	115 ± 23
+ CGS-21680 + ZM-241385	13 ± 4	52 ± 12

*K*_i, *High*_, D_2_R high-affinity value and *K*_i, *Low*_, D_2_R low-affinity value. Data are means ± SEM; *n* = 4, each determination performed at least in triplicate. Statistical analysis was performed by one-way ANOVA followed by the Tukey post hoc test. ***(*p* < 0.001); *significant increased* compared to vehicle. §§§(*p* < 0.001); *significant differences* compared to cells incubated with CHPG. †††(*p* < 0.001); *significant reduced* compared to cells incubated with CHPG and CGS-21680

In both HEK293T cells expressing D_2_R and mGluR_5_ or A_2A_R, D_2_R and mGluR_5_, the incubation with A_2A_R antagonist ZM-241385 (1 μM) and mGluR_5_ antagonist MPEP (300 μM) alone or in combination resulted in an almost complete blockade of the mGluR_5_ increase of the D_2_R *K*_i, *High*_ values and A_2A_R agonist-induced increase of mGluR_5_ agonist effects on the high-affinity D_2_R agonist binding sites (Tables 1 and 2).

### *cAMP Functional Experiments: the A*_*2A*_*R and mGluR*_*5*_* Protomers Interact and Modulate D*_*2*_*R Protomer Signalling*

**Fig. 5:**
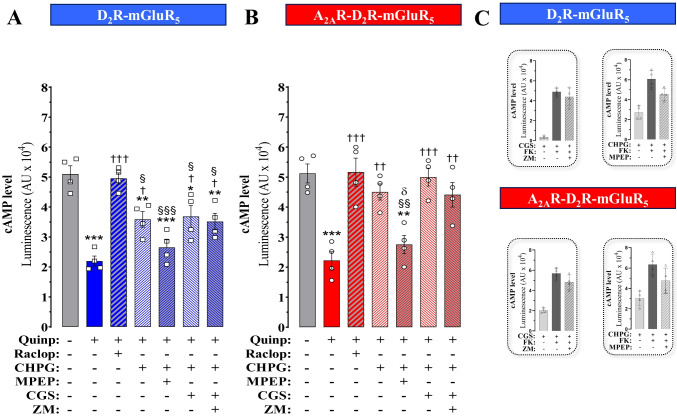
Functional evaluation of A_2A_R-mediated modulation of D_2_R-GluR_5_ heteromer. HEK293T cells transiently expressing D_2_R and mGluR_5_ (**A**) or A_2A_R, D_2_R and mGluR_5_ (**B**) were treated with forskolin before incubation with quinpirole (Quinp, X nM), raclopride (Raclop, X nM), CHPG (X nM), MPEP (X nM), CGS-21680 (CGS, X nM) and ZM-241385 (ZM, X nM). The cAMP levels were determined using a cAMP-Glo™ Assay detection kit (see Methods). Intracellular cAMP levels are given in luminescence intensity (AU, arbitrary units) after subtracting background basal luminescence (cAMP levels measured in non-transfected, non-treated cells). Results are expressed as means ± SEM; *n* = 4 independent experiments, each determination performed in quadruplicates. ***p < 0.001, **p < 0.01 and *p < 0.05, one-way ANOVA followed by Tukey’s post hoc test compared with cells treated only with forskolin (control). †††*p* < 0.001, ††*p* < 0.01 and †*p* < 0.05, one-way ANOVA followed by Tukey’s post hoc test when compared with cells treated only with quinpirole; §§§*p* < 0.001, §§*p* < 0.01 and §*p* < 0.05, when compared with cells treated only with quinpirole plus raclopride; δ*p* < 0.05, when compared to cells treated with quinpirole and CHPG. **C** CHPG and CGS21680-induced cAMP levels. HEK293T cells transiently expressing D_2_R and mGluR_5_ (top inset box) or A_2A_R, D_2_R and mGluR_5_ (Bottom inset box) were treated with and without forskolin before incubation with ligands. Results are expressed as means ± S.E.M.; *n* = 4 independent experiments, each determination performed in quadruplicates. Statistical analysis performed using one-way ANOVA followed by Tukey’s post hoc test (top inset box). CHPG versus CHPG + forskolin (*p* < 0.001), CHPG versus CHPG + forskolin + MPEP (*p* < 0.05); CGS-21680 versus CGS-21680 + forskolin (*p* < 0.001), CGS-21680 versus CGS-21680 + forskolin + ZM-241385 (*p* < 0.001) (bottom inset box). CHPG versus CHPG + forskolin (*p* < 0.01), CHPG versus CHPG + forskolin + MPEP (*ns*); CGS-21680 versus CGS-21680 + forskolin (*p* < 0.001), CGS-21680 versus CGS-21680 + forskolin + ZM-241385 (*p* < 0.001). Concentrations for ligands used: quinpirole (100 nM), forskolin (5 µM), raclopride (1 μM), CHPG (500 nM), MPEP (300 μM), CGS-21680 (100 nM) and ZM-241385(1 μM)

In cells expressing D_2_R and mGluR_5_ forming D_2_R-mGluR_5_ heterocomplexes (Fig. 2), the D_2_R agonist activation with quinpirole (100 nM) induced a G_i_ protein-mediated inhibition of adenylyl cyclase that first was raised with 5 µM forskolin (Fig. 5A). This effect was highly significantly blocked by the D_2_R antagonist raclopride (1 μM). In these cells, the mGluR_5_ agonist CHPG stimulation significantly counteracted the D_2_R agonist-induced reduction of cAMP accumulation (Fig. 5A). The significant effect of CHPG (500 nM) was significantly reduced by the mGluR_5_ antagonist MPEP (300 μM). The co-treatment with the A_2A_R agonist did not enhance the counteraction of the inhibitory D_2_R signalling by CHPG (Fig. 5A).

Likewise, quinpirole significantly reduced the cAMP level in cells expressing A_2A_R, D_2_R and mGluR_5_ (Fig. 5B). The mGluR_5_ agonist CHPG had an improved ability to counteract the adenylyl cyclase inhibition produced by the D_2_R agonist in these cells, yielding cAMP levels similar to those obtained after blocking D_2_R signalling with raclopride (Fig. 5B). Upon A_2A_R and mGluR_5_ agonist co-activation, a larger counteraction of the D_2_R agonist action was found compared to that obtained with such a co-treatment performed in cells expressing only D_2_R and mGluR_5_. These results suggest a synergistic and significant counteraction by A_2A_R and mGluR_5_ agonists of the D_2_R agonist-induced decrease of cAMP accumulation (Fig. 5B). Such effects of the combined agonist treatment were only weakly reduced by the A_2A_R antagonist (ZM-241385). In cells not expressing the A_2A_R, the A_2A_R antagonist failed to produce any changes in the cAMP accumulation under such co-agonist treatments.

It should be noted that CHPG agonist produces similar increases in cAMP levels as found after the A_2A_R agonist GGS in HEK293T cells co-expressing D_2_R, A_2A_R and mGluR_5_ (Fig. 5C). Therefore, we should consider also that mGluR_5_ might simply activate Gs, inducing cAMP accumulation, independently of D2-Gi-induced inhibition of adenylate cyclase (Fig. 5 A−C).

### Experiments on Haloperidol-Induced Catalepsy

Catalepsy is a nervous condition characterized by loss of muscle control and fixity of posture. It is considered a symptom of certain nervous disorders such as Parkinson’s diseases and epilepsy [44]. It is also a characteristic symptom of cocaine withdrawal, as well as one of the features of catatonia. The catalepsy is mainly produced by haloperidol induced blockade of D2R complexes in the dorsal striatal-pallidal GABA neurons within the dorsal striatum [44–46]. These GABA neurons mediate motor inhibition, counteracted by D_2_R agonist-induced activation of the D_2_R homo- and heterocomplexes like the D_2_R-A_2A_R or the D_2_R-mGluR_5_ heterocomplexes [24, 47–50]. The D_2_R activation of the dorsal striatal-pallidal GABA neurons is also essential for maintenance of normal locomotor activity.

**Fig. 6:**
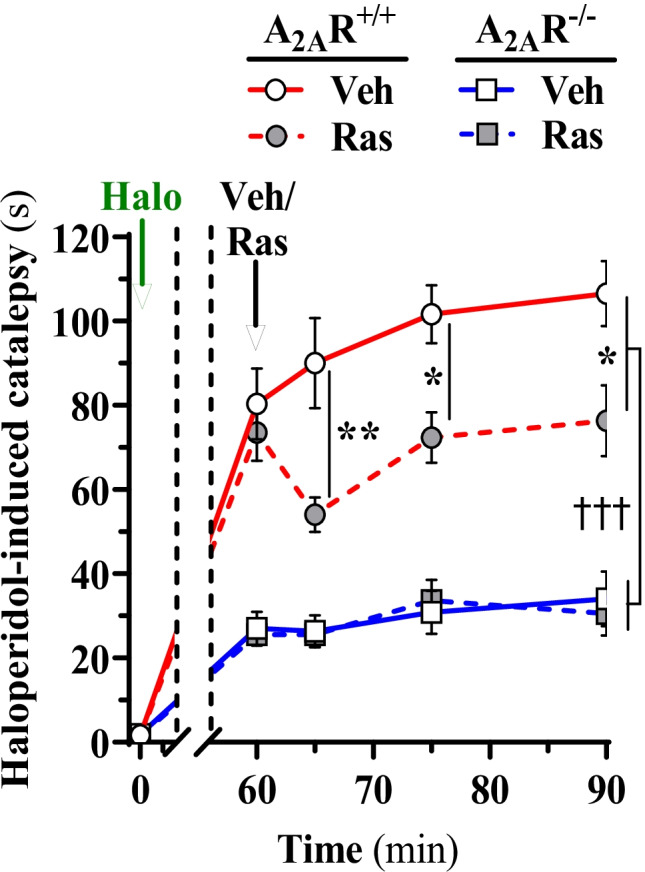
A_2A_R expression is needed for mGluR_5_ modulation of D_2_R-dependent behaviour in mice. Raseglurant reverses haloperidol-induced catalepsy. Haloperidol-induced cataleptic behaviour was measured as the time spent with both front paws resting on the bar (see Methods). Wild-type (circles), A_2A_R.^−/−^ (squares) animals were pretreated with haloperidol (i.p., 1 mg/kg, i.p.) at time 0 (green arrow). Subsequently, 1 h later, animals were administered (i.p.; black arrow) with either vehicle (20% DMSO in saline) or raseglurant (Ras, 1 mg/kg). The time spent in a cataleptic position was measured after 5, 15 and 30 min after raseglurant (or vehicle) administration. Results are expressed as the mean time spent cataleptic ± SEM over a period of 120-s measurement (*n* = 6 animals per group). The cataleptic behaviour was calculated and compared within groups by a multiple *t*-test statistical analysis. *p < 0.05 and **p < 0.01, two-way ANOVA followed by Šídák’s post hoc test. ††††*p* < 0.0001, three-way ANOVA (phenotype, F_(1, 100)_ = 279.6)

The catalepsy induced by the D_2_R antagonist haloperidol was evaluated in 10-min time intervals from 60 to 90 min after the injection of haloperidol (Fig. 6). In wild-type mice, the mGluR_5_-negative allosteric modulator raseglurant produced in this time period a significant reduction of the catalepsy time which was in the order of 25% (Fig. 6). In contrast, such a reduction of catalepsy was not observed by raseglurant treatment of A_2A_R^−/−^ mice. Furthermore, in vehicle-treated A_2A_R^−/−^ animals, the haloperidol-induced catalepsy was markedly reduced compared to that obtained in vehicle treated wild-type mice (Fig. 6).

## Discussion

The field of dopamine D_2_Rs changed markedly with the discovery of many types of D_2_R homo- and heteroreceptor complexes in subcortical limbic areas as well as the dorsal striatum [4, 16, 40]. The results indicate that the D_2_R is a hub receptor [51] which interacts not only with many other GPCRs including dopamine isoreceptors but also with ion-channel receptors, receptor tyrosine kinases, scaffolding proteins and dopamine transporters [24, 52, 53]. Disturbances in several of these D_2_R heteroreceptor complexes may contribute to the development of brain disorders through changes in the balance of diverse D_2_R homo- and heteroreceptor complexes mediating the dopamine signal, especially to the ventral striato-pallidal GABA pathway [37, 52, 54]. Of high relevance was the discovery of A_2A_R-D_2_R and A_2A_R-mGluR_5_ heteroreceptor complexes in native tissue [4, 16, 40, 55, 56]. Furthermore, the existence of the D_2_R-mGluR_5_ heterodimers in the biomembranes of living cells was demonstrated by bimolecular fluorescence complementation experiments in cellular models [21]. Although when tested by FRET microscopy in tsA 201 cells, D_2_R did not associate with mGluR_5_ [57]. Nevertheless, by combination of bimolecular fluorescence complementation and bioluminescence resonance energy transfer techniques, as well as the sequential resonance energy transfer technique, the occurrence of an A_2A_R-D_2_R-mGluR_5_ heteroreceptor complexes was observed in living cells. Furthermore, by co-immunoprecipitation, experiments validated the existence of an association of mGluR_5_, D_2_R and A_2A_R in rat striatum homogenates [21].

Herein, we present new findings that further expand the understanding of A_2A_R-D_2_R-mGluR_5_ heteroreceptor complexes. Also, strong evidences which support that the expression of the A_2A_R is necessary to facilitate the association of D_2_R and mGluR_5_ in a complex.

Our new findings are that transient co-expression of A_2A_R in HEK293T cells together with D_2_R^*Rluc*^ and mGluR_5_^*GFP2*^ resulted in a significant and marked increase in the formation of the D_2_R-mGluR_5_ heterodimer, a component of the A_2A_R-D_2_R-mGluR_5_ heterocomplex, based on the increase in the BRET^2^ max values. Such an increase could be related to the development of an increased affinity of the two D_2_R and mGluR_5_ protomers for each other due to allosteric changes related to the formation of the A_2A_R-D_2_R-mGluR_5_ complex. In line with this hypothesis, the BRET^2^50 values were significantly reduced for the D_2_R-mGluR_5_ heteromeric component of this trimeric heteroreceptor complex.

These results are also supported by the demonstration with PLA that an increased density of PLA-positive D_2_R-mGluR_5_ clusters was observed when A_2A_R expression had been added to the cells compared to cells only expressing D_2_R and mGluR_5_. In agreement, in the mouse dorsal striatum, the D_2_R-mGluR_5_ complexes were significantly reduced in the A_2A_R^−/−^ mice. Thus, it becomes clear that the expression of the A_2A_R in the mouse dorsal striatum is necessary to facilitate that the D_2_R and mGluR_5_ form a complex. It underlines that the multiple receptor protomers in the high-order heteroreceptor complexes are dependent on each other to improve or facilitate the formation of such complexes in the dorsal striatum.

The different results obtained on haloperidol-induced catalepsy in wild-type mice vs A_2A_R^−/−^ mice are of substantial interest since they can indicate a functional role of the A_2A_R-D_2_R-mGluR_5_ heteroreceptor complexes in the dorsal striatum as previously discussed [8, 17]. There was a marked reduction in the haloperidol-induced catalepsy in the A_2A_R^−/−^ mice compared to wild-type mice. Thus, in the absence of the A_2A_R, the D_2_R antagonist haloperidol appears to have a substantially reduced potency to block the D_2_R which can be caused by the loss of the antagonistic A_2A_R-D_2_R interaction [9, 58]. According to the current findings in cell lines, the D_2_R-mGluR_5_ heterocomplexes should be also formed to a much lower degree in the absence of A_2A_R in view of their dependency of A_2A_R according to the PLA experiments performed. The counteraction of the D_2_R-mediated inhibitory actions on cAMP signalling by CHPG, a mGluR_5_ agonist, was in our cell line also more effective in cells co-expressing beside D_2_R and mGluR_5_, also A_2A_R.

It seems likely that the formation of the A_2A_R-D_2_R-mGluR_5_ complex enhances the affinity of the D_2_R and mGluR_5_ protomers for each other in this complex. It is of high interest that the biochemical binding experiments reveal that the mGluR_5_ CHPG agonist-induced increase in D_2_R *K*_i, *High*_ values becomes significantly higher in the A_2A_R-D_2_R-mGluR_5_ complex compared to the D_2_R-mGluR_5_ complex despite the absence of A_2A_R agonist exposure. Thus, although agonist activation of the A_2A_R seems necessary to exert negative allosteric modulation of the D_2_R protomer agonist binding via heteroreceptor complexes, an increased constitutive activity of the A_2A_R protomer could explain the above results.

As expected, the combined incubation with CHPG and CGS-21680 led to an even stronger increase in the D_2_R *K*_i, *High*_ values of the A_2A_R-D_2_R-mGluR_5_ complex, demonstrating the impact of the A_2A_R protomer on the D_2_R-mGluR_5_ allosteric interactions, which can involve both constitutive and A_2A_R agonist-induced inhibition of D_2_R agonist binding. Our findings represent one of the first examples of integrative activity within a higher-order heteroreceptor complex and show how one receptor (A_2A_R) can substantially modulate the structure and recognition of a participating receptor heterodimer (D_2_R-mGluR_5_) in such a trimeric receptor complex.

The pharmacological analysis of the A_2A_R-D_2_R-mGluR_5_ complex and its impact on cAMP levels indicated that the A_2A_R can modulate the effects of the D_2_R-mGluR_5_ interactions on cAMP signalling. It was found that when the A_2A_R-D_2_R-mGluR_5_ complex was likely to be formed through the expression also of the A_2A_R, the mGluR_5_ agonist had an increased ability to counteract the D_2_ agonist-induced *G*_i/o_-mediated inhibition of the cAMP levels in comparison with the counteraction observed in the absence of A_2A_R expression. The same was also true for the combined treatment with the mGluR_5_ agonist CHPG and the A_2A_R agonist CGS-21680 when the A_2A_R was coexpressed. A stronger counteraction of the D_2_R-induced inhibition of the cAMP levels was observed when A_2A_R expression was present.

Taken together, our work on cell lines gives strong indications that, in the A_2A_R-D_2_R-mGluR_5_ complex, the A_2A_R protomer enhances the formation of the D_2_R-mGluR_5_ component of the complex with enhanced inhibition of D_2_R agonist binding recognition and its *G*_i/o_-mediated cAMP signalling. The inhibitory effects by A_2A_R and mGluR_5_ on D_2_R recognition and signalling reveal a significant molecular integration in A_2A_R-D_2_R-mGluR_5_ complexes, likely formed also in the dorsal striatum. The A_2A_R and mGluR_5_ antagonists targeting the A_2A_R-D_2_R-mGluR_5_ complexes in dorsal striatum may reduce the haloperidol-induced catalepsy by removal of the A_2A_R and mGluR_5_ protomer-mediated allosteric inhibition of the D_2_R protomer. Understanding of the trimeric complexes formed by these GPCRs could provide novel strategies for development of drugs against neuropsychiatric and neurodegenerative diseases by targeting their antagonistic receptor-receptor interactions.

## Supplementary Information

Below is the link to the electronic supplementary material.

Supplementary file1 (PPTX 18542 KB)

## Data Availability

The datasets generated during and/or analysed during the current study are available (upon request) in the Fuxe Lab repository at the Department of Neuroscience, Karolinska Institutet (contact email: Kjell.Fuxe@ki.se).
