# Supplementary figures and images for: The mGlu5 Receptor Protomer-Mediated Dopamine D2 Receptor Trans-Inhibition Is Dependent on the Adenosine A2A Receptor Protomer: Implications for Parkinson’s Disease
Source: Mol Neurobiol. 2022 Jul 12;59(10):5955–69. doi: 10.1007/s12035-022-02946-9 (PMC9463353; doi:10.1007/s12035-022-02946-9)

## Slide 1
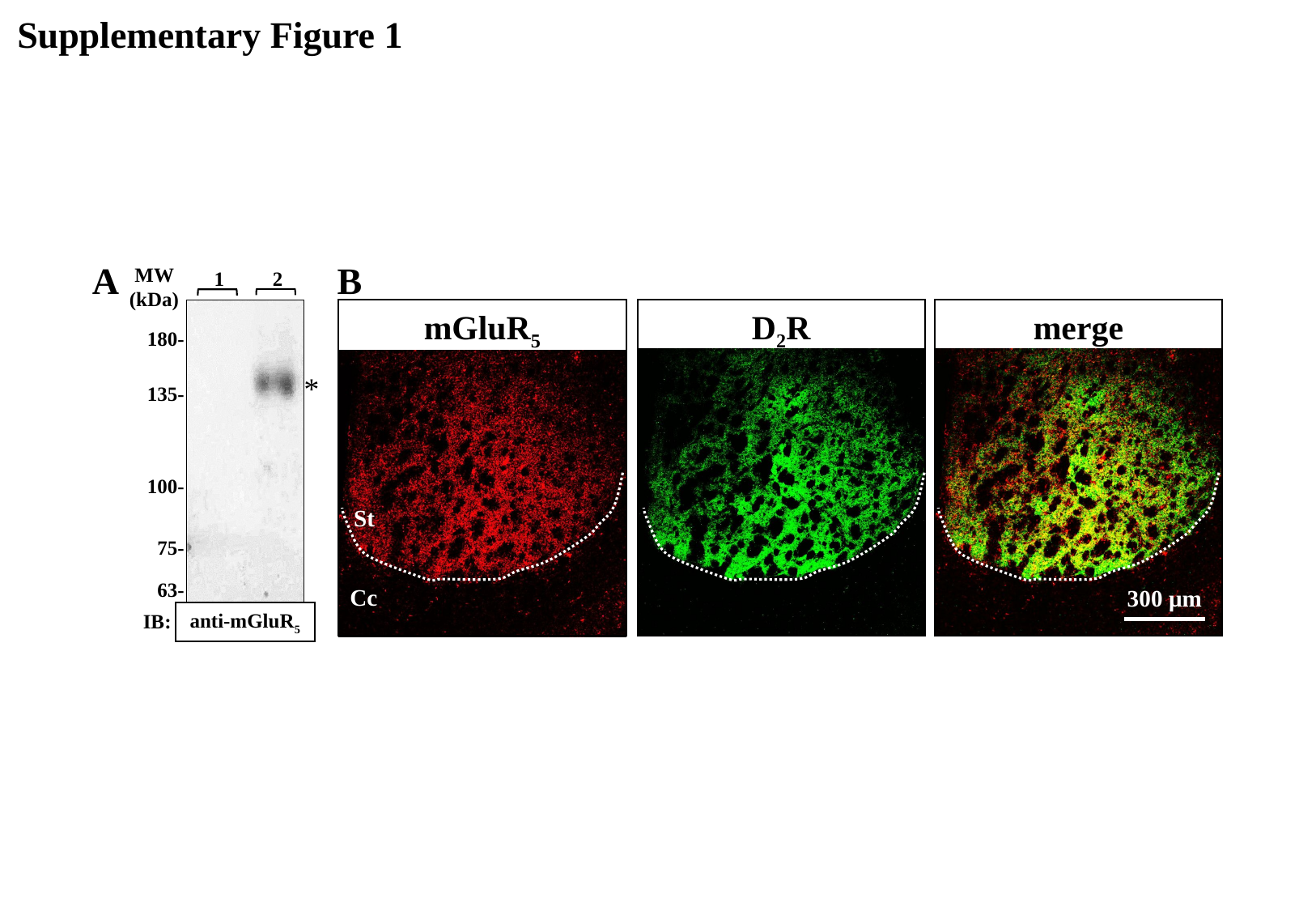

Supplementary Figure 1
A
B
MW
(kDa)
1
2
D2R
mGluR5
merge
180-
*
135-
100-
St
75-
63-
Cc
300 μm
IB:
anti-mGluR5

Supplement: Supplementary file 1 — Supplementary file1 (PPTX 18542 KB) [file 12035_2022_2946_MOESM1_ESM.pptx]
